# A Wrist System for Daily Stress Monitoring Using Mid-Level Physiological Fusion and Late Fusion with Survey-Based Labels

**DOI:** 10.3390/s25216592

**Published:** 2025-10-26

**Authors:** Marija Simic, Sravya Reddy Yammanuru, Geraline Saguiafin, Jananan Velvelicham, Sridhar Krishnan

**Affiliations:** Signal Analysis Research (SAR) Group, Department of Electrical, Computer, and Biomedical Engineering, Toronto Metropolitan University (TMU), 350 Victoria Street, Toronto, ON M5B 2K3, Canada; sravyareddy0205@gmail.com (S.R.Y.); gsaguiafin@torontomu.ca (G.S.); jvelvelicham@torontomu.ca (J.V.); krishnan@torontomu.ca (S.K.)

**Keywords:** multi-sensor fusion, galvanic skin response, heart rate variability, skin temperature, SpO_2_, Bluetooth Low Energy, time alignment, questionnaires, wearables, stress monitoring

## Abstract

**Highlights:**

**What are the main findings?**

- Multi-sensor fusion of physiological data (GSR, HRV, temperature, SpO_2_) with self-reported labels achieved a Mean Squared Error (MSE) of 0.08 on stress prediction, demonstrating the technical feasibility of the wrist system.
- The proposed wrist system’s SpO_2_ and Beats Per Minute (BPM) measurements show high accuracy, tracking a commercial benchmark with a Mean Absolute Error (MAE) of 0.23 for SpO_2_ and 4.94 for BPM.

**What is the implication of the main findings?**

- Wrist temperature (r = −0.43) and Heart Rate Variability (HRV) (r = 0.36) were identified as the strongest and most complementary physiological features for stress, guiding future development of multi-modal stress monitors.
- The mid-level physiological fusion combined with late fusion of user self-reports provides a stronger and context-aware approach to stress monitoring than systems relying solely on physiological or self-report data alone, as it better aligns predictions with the user’s perception of stress.

**Abstract:**

Multi-sensor fusion can improve daily stress monitoring. Methods: A wrist-worn device includes a system of the galvanic skin response (GSR), PPG-derived heart rate variability (HRV), skin temperature, and SpO_2_, paired with self-reported questionnaires. The device streams data to a mobile app over Bluetooth Low Energy and updates the UI within 1–2 s. The physiological features are captured within a fixed window around each questionnaire time and undergo a mid-level fusion; late fusion is also evaluated using self-reports. Results: Against a commercial reference device, the proposed system achieved a mean absolute error of 0.23 for SpO_2_ and 4.94 for BPM in a one-day benchmark session. The system was validated through a technical evaluation using representative inputs and simulated survey labels. The fusion model was evaluated using simulated physiological and survey data. Using a support vector machine algorithm, a mean squared error of 0.08 was achieved when predicting simulated stress labels. Temperature was shown to have the strongest correlation with simulated stress levels at −0.43, followed by heart rate variability (HRV) at 0.36, while SpO_2_ had a negligible correlation at 0.09 in the current dataset. Conclusion: The system integrates multi-sensing, on-device preprocessing, BLE transmission, and a clear fusion workflow that creates a useful predictive performance of daily stress monitoring.

## 1. Introduction and Background

Due to the many responsibilities and obligations placed upon students, university life can often be described as a time of great stress. Students face academic pressure from exams and assignments, along with grade maintenance. Some students face challenges with relocating and adapting to a new environment, adjusting to new social norms, and forming new connections. Lifestyle changes in food, exercise, sleep patterns, and time management can add strain. Additionally, financial pressures such as student loans, tuition costs, and part-time employment may be a significant burden. More than 80% of students report experiencing stress during their academic careers [1]. There has been a 30% increase in mental health conditions such as anxiety and depression among university students over the past decade [2], and 25% of dropouts due to mental health were reported in 2022 [1].

Stress may affect various aspects of life, which is why it is imperative to monitor and control it. Academic performance within university students may decline as cognitive function, memory, and decision-making are impaired [3]. Not only is mental health impacted, but stress has a substantial impact on physical health as well, including a weakened immune system; sleep disorders; and, in severe cases, cardiovascular problems [3]. Between negative academic and physical effects, stress diminishes overall well-being, often leading to burnout, a lower quality of life, and ineffective coping mechanisms. Early detection of stress allows for better management, which is essential to mitigate these harmful effects.

Current wrist-worn devices use temporal fusions, such as Kalman filtering, to improve stress classification [4]. However, such systems remain constrained to only physiological modalities and do not incorporate user-reposted (active) data in the fusion process [4]. In contrast, traditional methods for monitoring stress, involving self-report questionnaires and surveys, often fall short in accuracy as they rely on subjective input. Surveys such as the Perceived Stress Scale (PSS), Generalized Anxiety Disorder assessment (GAD-7), and Patient Health Questionnaire (PHQ-9) are commonly used, yet these surveys alone can underreport and are subject to bias.

To address the limitations of self-report and stress monitors, this work combines standardized assessments with physiological data from a wrist-worn device that measures heart rate variability (HRV), galvanic skin response (GSR), body temperature, and blood oxygen levels in a non-invasive and continuous manner to enable proactive monitoring and earlier management of stress. The proposed system uses mid-level fusion of the physiological data and late fusion with survey-based labels, enabling a stronger bridge between objective and subjective stress measures.

Section 2 describes the device hardware, sensing, signal processing, questionnaires, and fusion methods. Section 3 reports benchmarks and fusion results. Section 4 discusses limitations. Section 5 concludes.

## 2. Materials and Methods

For consistency, we use the abbreviations GSR (galvanic skin response), HRV (heart rate variability), and SpO_2_ (oxygen saturation).

### 2.1. Ethics Disclaimer

No identifiable human subject data was collected; the data collected was all simulated/self-test data for algorithm validation only. As such, research ethics board (REB) approval was not required.

### 2.2. Device Hardware and Architecture

The wearable device sits comfortably on the wrist, consisting of a GSR, PPG, body temperature, and blood oxygen sensor, which measure the physiological biomarkers of stress in a non-invasive and continuous manner. The device is designed for comfort and long-term wear. The system includes a mobile platform application to which data is streamed with BLE and then processed and visualized.

**Figure 1:**
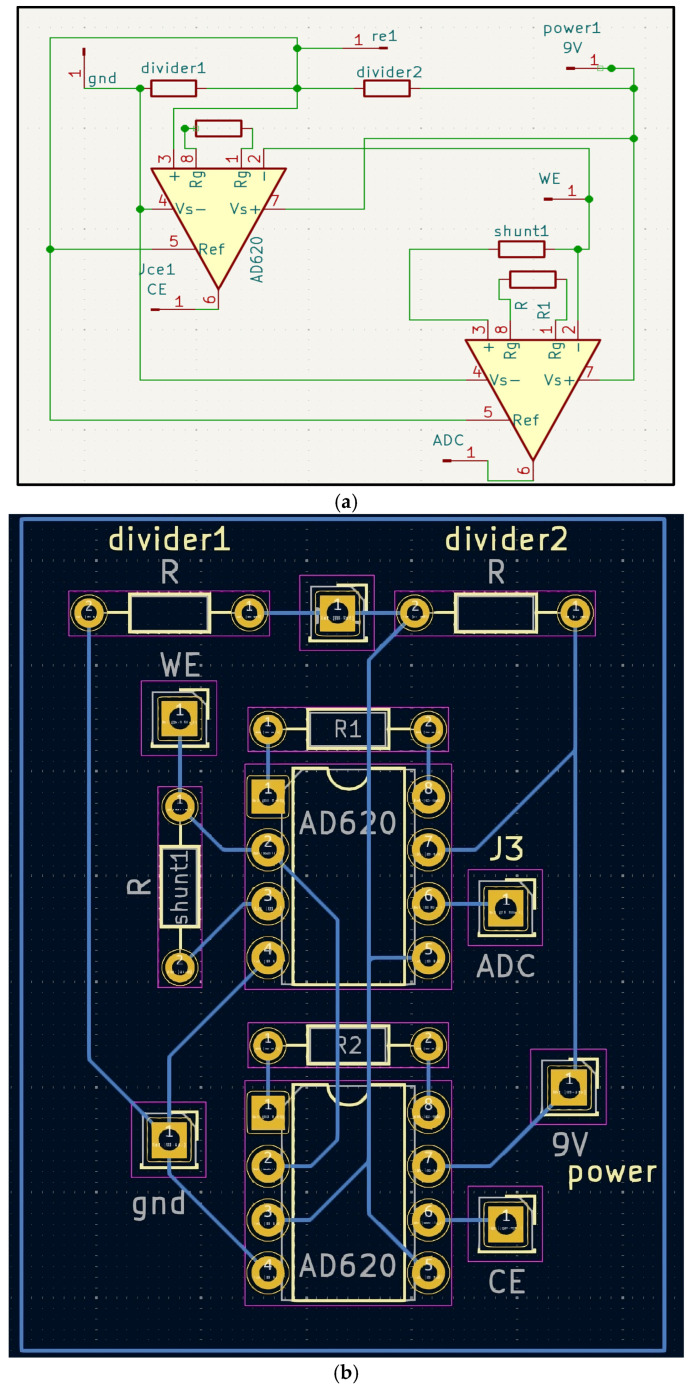
(**a**,**b**). Three-electrode GRS front-end. (**a**) Schematic showing working and counter electrodes (gold–graphene) and the reference electrode (Ag/AgCl). (**b**) One-layer PCB layout with component labels. The circuit holds a constant potential between the working and reference electrodes and measures current through the counter electrode as a proxy for skin conductance. R1, R2, divider1, and divider2 are resistors with a value of 10k ohms. R (shunt) has a resistor value of 100 ohms. WE is the pin for the working electrode, CE is the pin for the counter electrode, and RE is the pin for the reference electrode. Wire connects from this pin directly onto the corresponding PET substrate.

Galvanic skin response (GSR) front end. Advances in biosensing have focused on leveraging and using gold and graphene as the sensing materials for GSR due to their superior electrical properties, biocompatibility, and mechanical properties. A gold and graphene mixture combined in a three-electrode electrochemical system, with the working and counter electrode being the gold and graphene mixture, and the reference electrode being a silver or silver chloride [5]. The design of the GSR sensor is influenced by “eSkin” [5]; the exact ink, a gold and graphite mixture, was replicated for the working and counter electrode. To create the ink’s base, 4 g of graphite powder was mixed with 2 g of varnish. Edible gold leaves were crushed into a fine powder using a mortar and pestle. Gold leaf was added in the amount equivalent to 5% by mass of the base mixture. An amount of 5 mL of acetone was added to the mixture and then stirred for 12–15 min on a hot plate at 85 °C until a paste-like consistency was formed. The paste was then placed on a flexible PET (polyethylene terephthalate) substrate to form the counter and working electrodes. A conductive silver-chloride (Ag/AgCl) paste was also placed on a PET substrate to be the reference electrode. The gold and graphene mixture is sensitive to changes in the skin’s moisture; therefore, it can detect minor electrochemical fluctuations due to ion concentration shifts [6]. The working electrode will detect these changes, the reference electrode provides a stable potential reference, and the counter electrode completes the circuit by accepting the current from the working electrode through the skin [6]. The potential between the working and reference electrode is held at a constant, and the current between the working and counter electrodes is monitored and measured, reflecting the skin’s conductance. The PCB (Figure 1a,b) was designed as a single integrated board to reduce noise and improve wearability.

PPG and SpO_2_ module. The photoplethysmography (PPG) sensor, MAX86176/MAX30005 (Analog Devices, formerly Maxim Integrated, sourced from Wilmington, MA, USA), on the wrist records green and infrared reflectance to estimate the inter-beat intervals for the HRV and to compute the SpO_2_.

**Figure 2:**
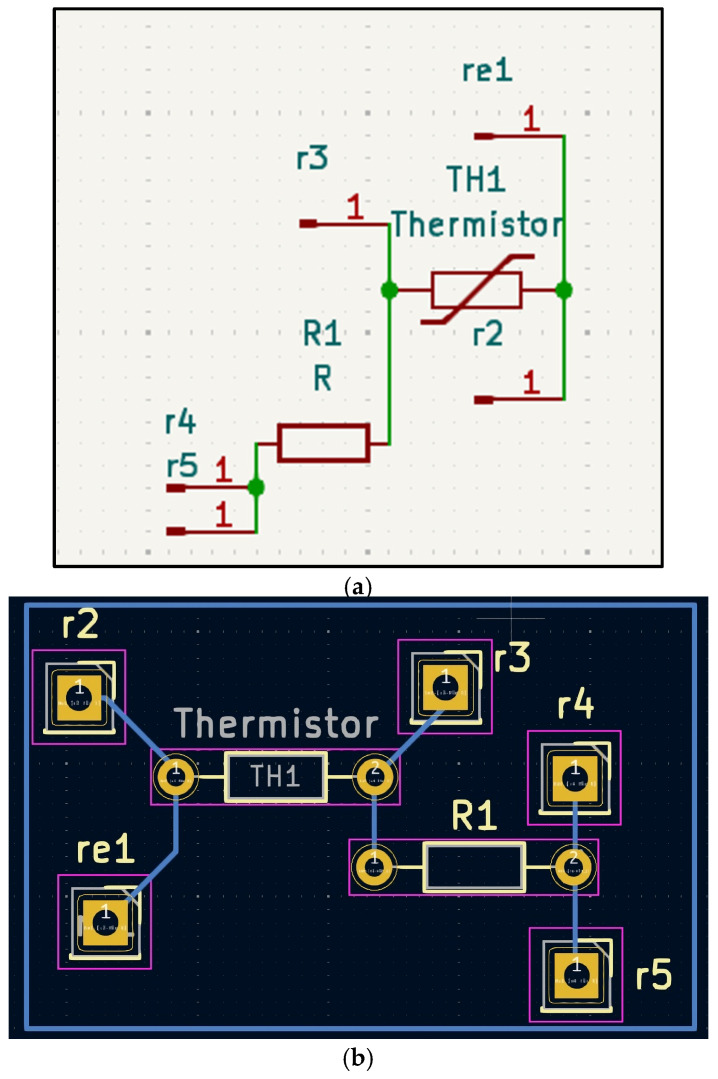
(**a**,**b**). Wrist-temperature module. (**a**) Contact thermistor schematic. (**b**) PCB layout and connector mapping. TH1 represents the thermistor; R1 is a resistor of 10k ohms; and r1, r2, r3, r4, and r5 are connector pins.

Skin-temperature module. A contact sensor was created using a thermistor to measure the wrist surface temperature for slow-varying changes (Figure 2a,b).

**Figure 3:**
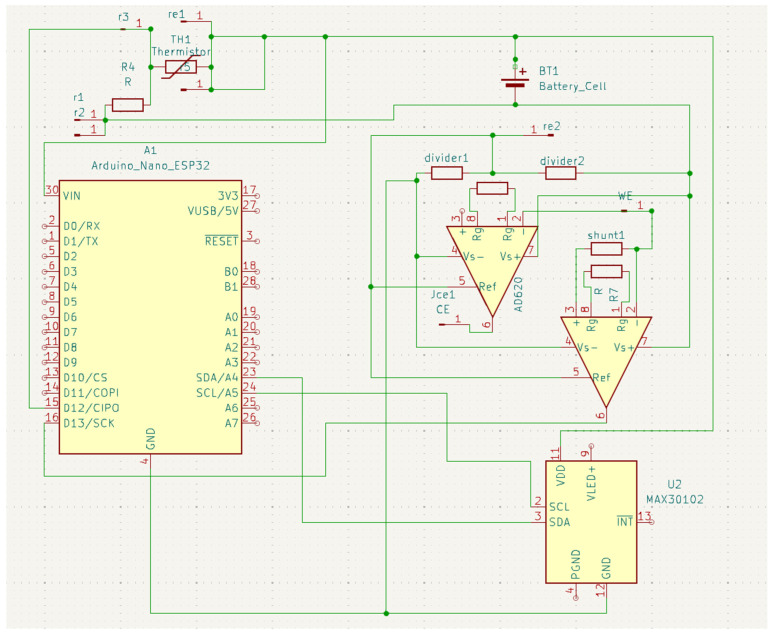
Schematics and layout of the display, GSR, PPG, and body temperature sensor.

**Figure 4:**
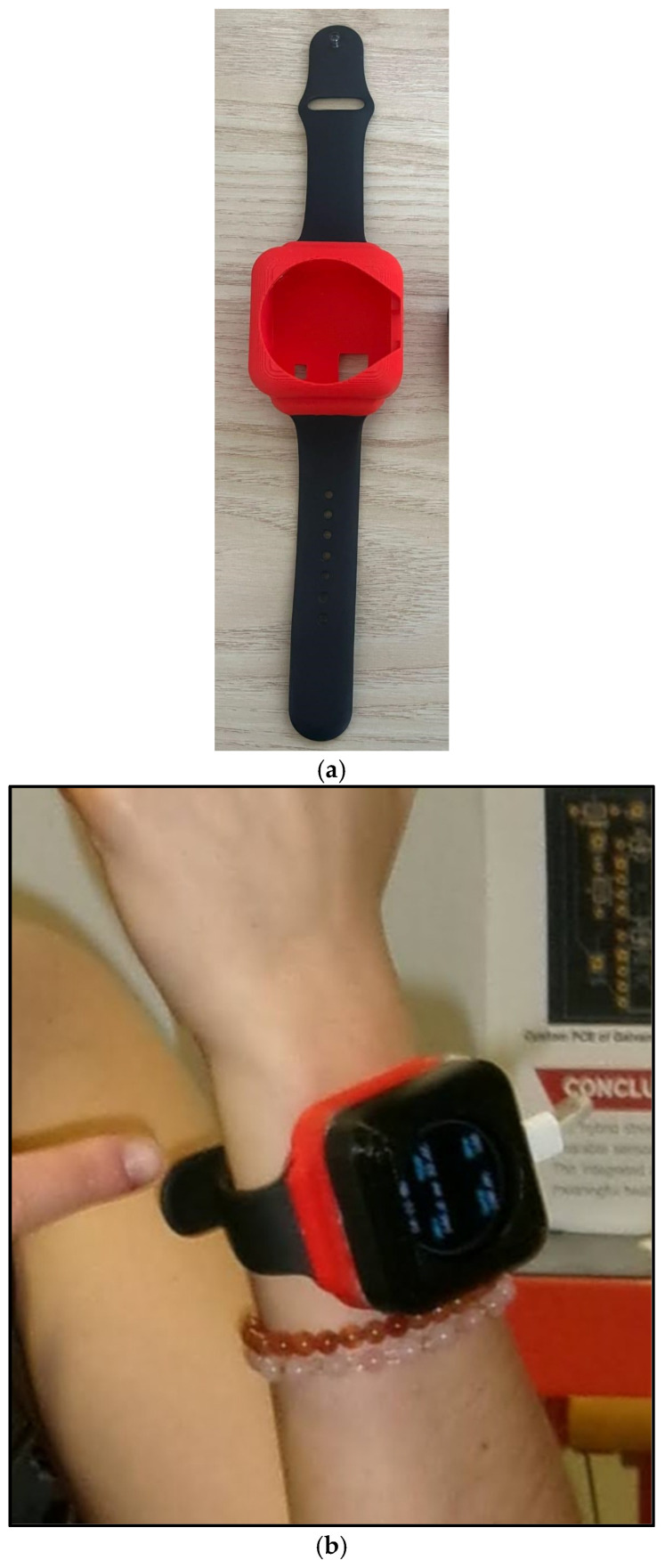
(**a**) The case of the device was 3D printed using a Bambu Printer, and the watch straps were added. (**b**) The integrated device worn by a human, which gives live results on the HRV, temperature, blood oxygen levels, and heart rate.

All sensors are connected to the analog-to-digital converters (ADCs) on the MCU through their respective front ends (Figure 3). The MCU filters and buffers the data before streaming it over BLE. The MCU also manages the duty cycle and sampling rates to reduce power while preserving the signal and aligns the sensor windows with the self-reports for downstreaming analysis. Power management was achieved through a dual-rail system: a boost converter (MT3608) generated 9 V for the GSR circuit, while the MCU and the other sensor operated at 3.3 V. As this work presents a first iteration of the prototype, a comprehensive battery life test was not performed. However, we have implemented several optimizations that vary the system’s power consumption across different operational modes, which are managed by the firmware: continuous mode, active mode, and communication mode. Continuous mode is for the skin temperature; it is sampled at 1 Hz, which has a very low power draw. Active mode controls the PPG sensor and the GSR. It runs for 60 s every 5 min at rest and for 120 s when the survey is being completed. Communication mode sends data over Bluetooth Low Energy (BLE) at 1–2 s intervals. It uses notifications with a connection interval of 60 to 90 ms. The data is batched into compact packets before transmission. The MCU and all sensors are compacted into a wrist-worn device (Figure 4a,b).

### 2.3. Sensor and Acquisition

Stress triggers many physiological responses as it activates the body’s fight or flight response. These physiological biomarkers, such as GSR, HRV, body temperature, and blood oxygen level, can be objectively measured. The non-invasive measurement of these biomarkers allows for them to be monitored continuously and provides insights into the body’s response to stress.

#### 2.3.1. Galvanic Skin Response (GSR)

The GSR, also referred to as the skin’s electrodermal activity, measures the changes in the skin’s electrical conductance [5]. The skin’s electrical conductance varies with the body’s sweat gland activity, which is regulated by the sympathetic nervous system [7,8]. When the body and mind are under stress, typically, the sympathetic nervous system is activated, leading to increased sweating even in minuscule amounts, altering the skin’s conductance. GSR is a reliable, non-invasive biomarker for stress detection. GSR is often combined with heart rate and body temperature monitoring for enhanced accuracy and monitoring. Studies have shown that under stressed scenarios, participants’ GSR increases by 100 to 400 percent [9,10]. Resting GSRs are measured to be around 0.9 to 1.1 μS (microsiemens), and under cognitive stress tasks and timed math problems and audio interference, the participants’ GSRs increased to 3.2 μS average and 2.5 to 4 μS [9,10].

#### 2.3.2. Heart Rate Variability (HRV)

HRV refers to the variations in time between consecutive heartbeats, measured by the R-R or N-N intervals, which are regulated by the autonomic nervous system. HRV reflects the dynamic relationship between the parasympathetic rest and digest and sympathetic fight or flight systems [7]. When stress is induced on the body, the sympathetic nervous system activity increases, whereas the parasympathetic activity is suppressed, reducing HRV [7]. In the time domain, HRV can be measured using the standard deviation of NN intervals and the root mean square of successive differences. Acute stress-induced scenarios can reduce an individual’s SDNN by 20 to 30 percent and RMSSD by up to 40 percent, whereas chronic stress can lead to consistent reductions exceeding 25 percent [11,12].

#### 2.3.3. Skin Temperature

The body’s thermoregulation can be altered due to stress, causing it to increase subtly. Since stress activates the sympathetic nervous system, it influences vasodilation and metabolic heat production as well [13]. Although core temperature can rise slightly due to blood flow redistribution, peripheral skin temperature actually decreases during acute stress [7]. Vasoconstriction at the wrist during a stress event may lead to a drop of 1 to 3 °C [9]. The body prioritizes the thermal regulation of the core, which causes a decrease in peripheral temperature [7].

#### 2.3.4. Blood Oxygen Saturation

An individual’s blood oxygen levels are determined by respiratory patterns. Stress may alter respiratory patterns due to hyperventilation or shallow breathing, which can be experienced during stressful events [12]. Hyperventilation and shallow breathing lead to reduced oxygen exchange efficiency, which then lowers blood oxygen saturation [12]. Changes in blood oxygen levels are evident in individuals who experience stress-induced anxiety or panic attacks [12].

### 2.4. Signal Processing Pipelines

Raw signals are transformed into features suitable for modeling. For HRV we use inter-beat intervals derived from the wrist PPG, and for GSR, temperature, and SpO_2_ we use continuous time series collected on the wrist. Feature extraction and filtering steps are applied consistently across participants.

#### PPG Signal Filtration Process

DC block filter (high-pass filter). The first step in this filtering process is the DC block filter, also known as a high-pass filter. This filter is designed to remove the DC offset from the raw PPG signal. The DC component represents the steady part of light absorption, which comes from the constant tissue absorption, skin tone, or ambient light. It is not really used for heart rate or variability detection, because it does not carry pulsatile information. The filter uses a formula that subtracts the previous input and includes a weighted portion of the previous output, canceling out slow-changing values. The formula used is y [n] = x [n] − x [n − 1] + a ∗ y [n − 1], where a is a constant, 0.95, which controls how much of the previous output is retained. The result of this filter is a signal that contains the AC component, which is the actual heartbeat waveform. This step is required as raw PPG contains strong DC components and needs to be removed to make the filtering process effective. The cutoff used is low (~0.3 Hz) so that only very slow drifts are removed, while the physiological heart rate band (0.5–5 Hz) is preserved.

Low-pass filter. After removing the DC offset, the signal still contains high-frequency noise, such as electrical interference, sensor jitter, or irrelevant signal fluctuations. The output of the DC block filter is passed through a low-pass filter, which smooths out the signal by averaging it over time. It retains slow, meaningful changes and discards rapid and irrelevant changes. The formula for this filter is y [n] = y [n − 1] + a ∗ (x [n] − y [n − 1]), where a is a constant, 0.1, that shows how responsive the smoothing is. A smaller one results in heavier smoothing. The output of this step is a clean, smooth waveform that shows the pulsatile signal with minimal noise. Its cutoff is selected around 8–10 Hz, which is above the maximum heart rate frequency but below typical noise sources, making sure that the pulsatile component is retained while high-frequency jitter and sensor noise are removed.

Combined Filtering (filter_sample Function). Both filters are combined into a single method called filter_sample. When a raw sample is passed through the function, it first applies the DC block to remove the baseline drift, and then a low-pass filter is applied to smooth the result. This two-step process makes sure that the output signal is both centered on zero (no DC) and free from jitter and high-frequency noise. The resulting filtered signal is ideal for detecting heartbeats accurately and consistently. This clean signal is used downstream for detecting peaks, calculating inter-beat intervals (IBIs), estimating heart rate (BPM) and HRV, and even computing SpO_2_ based on the red and infrared signal components. The order of applying the high-pass and then low-pass is important. First, remove the slow baseline drift, then smooth out fast noise.

### 2.5. Firmware and BLE Protocol

The system includes a mobile platform application, written in JavaScript, which serves as the user interface. The application prompts users to answer the mental health assessment surveys, displaying their real-time stress metrics, trends, and wellness scores. The device communicates sensor readings to the application for storage and visualization. The application links each survey response to the surrounding sensor data window for later analysis. The firmware was programmed on the ESP32 microcontroller in Arduino. This microcontroller was chosen for its integrated BLE module and processing power to handle on-device signal processing. The firmware architecture manages the sensor’s initialization, time synchronization, and pre-processing signals (HRV, SpO_2_, GSR, and temperature). It controls the sensor’s duty cycles and sampling rates. The raw data is filtered and downsampled on the device to reduce latency before BLE transmission. The MCU aligns the sensor data windows with the survey times for downstreaming the analysis and fusion. The firmware handles BLE protocol, it compacts the data, and the packets are transmitted to the mobile application at 1–2 s intervals, each containing the time-stamped features. The firmware design allows for on-device data reduction, which is crucial for power optimizing and ensuring long-term usability.

### 2.6. Data Fusion Strategy

The system employs a machine learning algorithm to accurately identify stress by processing and analyzing physiological data and fusing it with active data to enhance accuracy. We use a time-aligned approach that pairs each self-report with the surrounding passive window. For each self-report questionnaire submitted at a time $t_{s}$, a systematic window is formed with Δ=15 min. For each window, the features for HRV, GSR, skin temperature, and SpO_2_ are computed. The self-report $t_{s}$ provides a label for that window. Mid-level fusion includes mapping each modality to a compact embedding, concatenating embeddings, and fitting to a regressor. Late fusion uses the self-reports and combines them with the physiological prediction using a weighted equation: $y=\alpha y_{s}+(1-\alpha )s$, where s is the normalized survey score, $y_{s}$ is the physiological prediction, and α is the weight selected through cross-validation.

**Figure 5:**
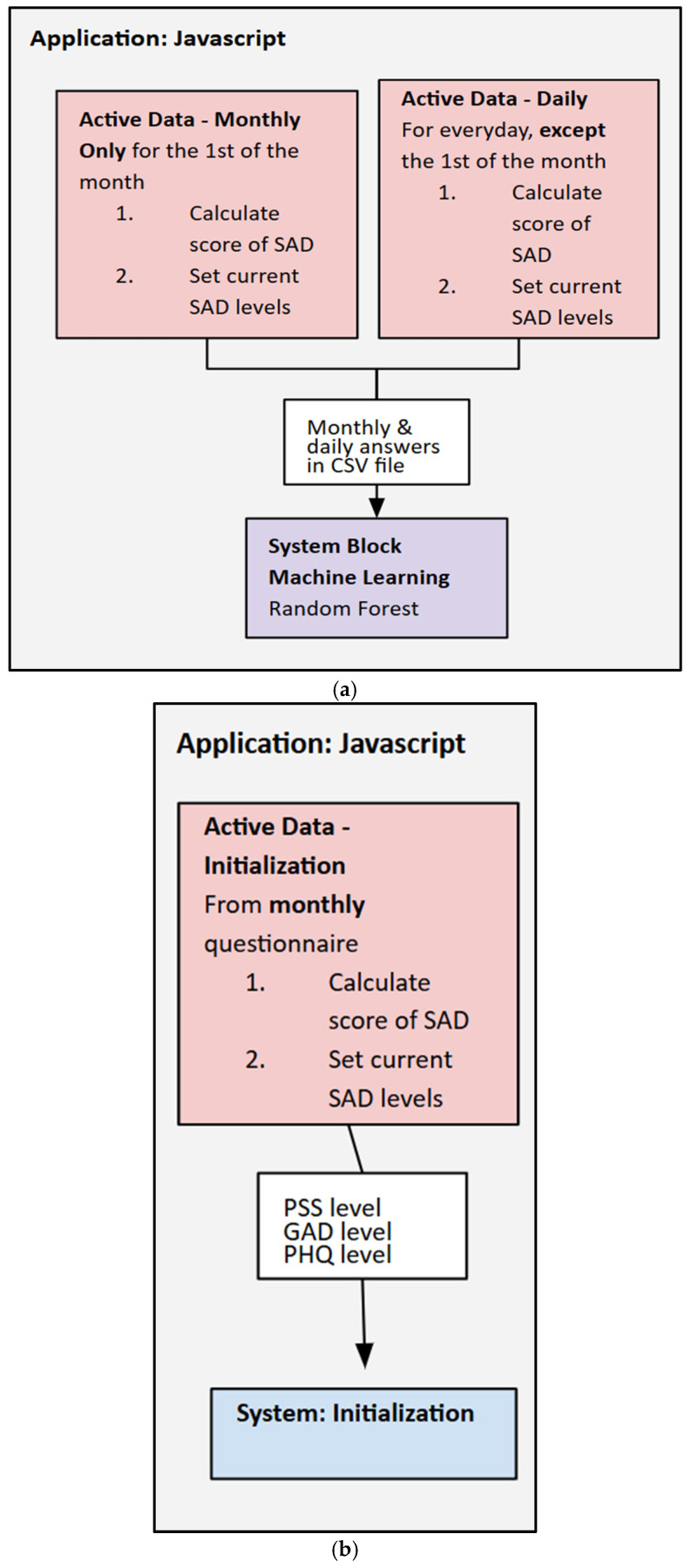

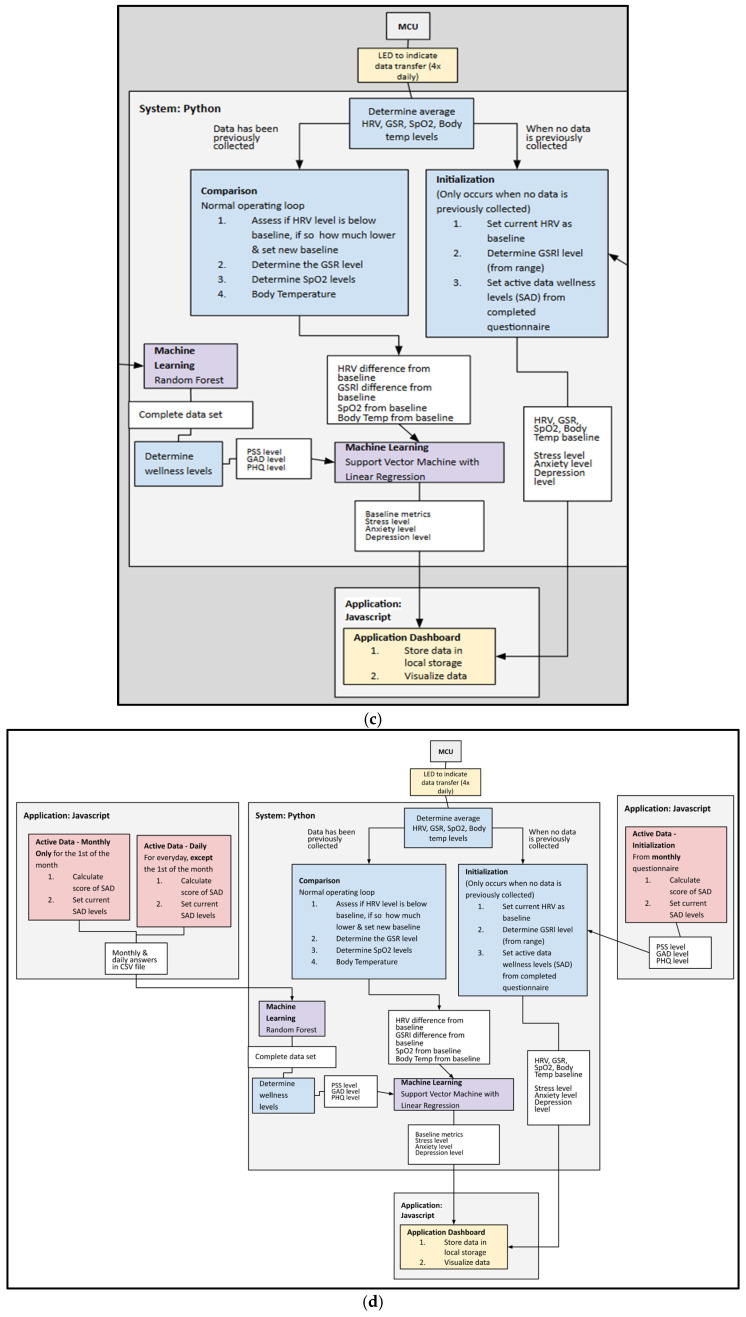
(**a**–**d**). Device system flowchart, the operational architecture for wellness and stress level assessment. Components include (**a**) the monthly and daily active data (JavaScript ES2024) calculation; (**b**) the initialization for determining baseline and daily wellness metrics; (**c**) machine learning model (random forest and support vector machine) operating loop for predicting wellness; and (**d**) the overall system flow, including data transfer via the MCU and visualization via the application dashboard (JavaScript).

For mid-fusion, an SVM and a linear regression model were used to combine the passive data. Each of the four physiological inputs, HRV, body temperature, SpO_2_, and GSR, is first processed individually. Each scalar input uses a fully connected layer as an encoder to map its value into a compact embedding vector. Each embedding has a size of 4 levels, which linearizes the input. The embeddings are then concatenated to form a single, fused feature vector of a size of 16 levels (4 modalities × 4 dimensions). This fused vector is then passed through another fully connected layer leading to the final output for wellness/stress prediction (see Figure 5a–d). The HRV weekly averages were selected to be the primary input due to their strong correlation to reported stress levels. Embedding the passive and active features into the SVM and using a simple weight to improve accuracy allowed the model to learn a nonlinear relationship between the features and perceived stress.

### 2.7. Power and Resource Optimization

The device is optimized for high compliance, as well as long-term usability and durability. At rest, the PPG and GSR run for 60 s every 5 min. During the completion of the self-report, the sensors run for 120 s. The skin temperature is sampled at 1 Hz continuously, given its dynamics. The PPG is sampled at 25 Hz, and the GSR has a sampling rate of 10 Hz. The HRV is computed from the 60–120 s segments to stabilize the metrics.

The BLE uses notifications with a connection interval of 60 to 90 ms, a slave latency of 3 to 5, and a supervision timeout of 4 to 6 s. The sampled data is batched into compact packets before transmission, maintaining around 1 to 2 s phone UI latency with a low average current.

### 2.8. Questionnaires and Labeling

This work uses publicly available survey frameworks, such as the Perceived Stress Scale (PSS), Generalized Anxiety Disorder Assessment (GAD-7), and Patient Health Questionnaire-9 (PHQ-9), to generate simulated labels for evaluating our algorithm’s performance. These frameworks are standardized tools used by doctors and clinicians to evaluate the psychological state. They were adapted and implemented here for a technical proof-of-concept study to test the alignments between the device features and subjective rating, not for clinical diagnosis. Because of the stigma associated with mental health, these self-reporting frameworks are prone to underreporting as due to social stigma or subjective bias [13,14]. For this reason, this technical evaluation adapted the framework as a means of simulating a labeled dataset for algorithm development.

Perceived Stress Scale (PSS). The PSS scale measures stress by presenting 10 questions that need to be scored on a 5-point Likert scale. For the daily assessment of stress, this was narrowed down to 3 questions to promote daily completion. The scale ranges from 0, never; 1, almost never; 2, sometimes; 3, fairly often; and 4, very often. The score is calculated by summing up the points for each question, with a scoring range of 0 to 15. For this study, the levels have been divided into the following categories: minimal stress, 0 to 4; mild stress, 4 to 8; moderate stress, 8 to 12; and severe stress, 12 to 15. The PSS assessment has sensitivity rates ranging from 70 percent to 90 percent, with specificity rates between 75 percent and 85 percent, demonstrating good construct validity. Although the assessment is effective for tracking perceived stress and evaluating the need for intervention, it is not a diagnostic tool [14].

Generalized Anxiety Disorder Assessment (GAD-7). The GAD-7 assessment is used to assess the severity of generalized anxiety. The assessment consists of 7 questions that are to be answered on the 4-point scale. For the daily assessment of anxiety, this was narrowed down to 3 questions to promote daily completion, as well as altered to be answered on a 5-point scale for consistency with assessing stress. The total score is calculated by summing up all the points from the questionnaire and dividing up the score into four categories: minimal anxiety, 0 to 4; mild anxiety, 4 to 8; moderate anxiety, 8 to 12; and severe anxiety, 12 to 15. The sensitivity range for the GAD-7 assessment ranges from 76 percent to 89 percent, with specificity rates between 82 percent and 95 percent. Although the GAD-7 assessment is not a diagnostic tool, it is effective in identifying and evaluating the symptoms of generalized anxiety [15].

Patient Health Questionnaire-9 (PHQ-9). PHQ-9 is an assessment tool used to measure the severity of depressive symptoms. The assessment consists of 9 questions scored on a 4-point scale. For the daily assessment of depression, this was narrowed down to 3 questions to promote daily completion, as well as altered to be answered on a 5-point scale for consistency with assessing stress and anxiety. The total score is summed up and categorized in one of the following: minimal depression, 0 to 3; mild depression, 3 to 6; moderate depression, 6 to 9; and severe depression, 9 to 12. The PHQ-9 assessment has a sensitivity rate of 88 percent and a specificity rate of 85 percent. The assessment is a valuable tool for clinicians to monitor patients who suffer from depression, but it should not be used as a diagnostic tool [15,16].

In the study, abbreviated versions of the questionnaires were used to minimize the burden on the user and ensure high completion rates. To ensure the validity of the abbreviated questionnaire, items were selected that would best represent the core constructs of the questionnaires while reducing the length. From each original questionnaire, specific questions were chosen to maintain the validity of the abbreviated version. For instance, the abbreviated PSS questionnaire includes a question that captures both the feeling of being overwhelmed and lack of control, which are two questions in the full PSS questionnaire. Although the simulated data precluded the ability to perform a psychometric validation, the careful selection of the key items ensures that the framework of the abbreviated questionnaire aligns with the original validated scales.

Label binding. Each simulated label generated from the adapted questionnaire framework is used as a data point for the corresponding time period. These labels are then linked to the surrounding passive data for analysis. The questionnaire contains 4 questions, where each question has 5 severity levels. The questions are divided as follows: one question that pertains to both PSS and GAD, one question that pertains to GAD, one question that pertains to both PSS and PHQ, and one question that pertains to PHQ. After the questionnaire is answered, the score is evaluated using a random forest. Along with this score, the values of HRV, temperature, and SpO_2_ are analyzed using a support vector machine (SVM) to provide the stress meter reading. The severity of the stress is segregated into 4 different levels: minimal, mild, moderate, and severe.

## 3. Results

### 3.1. Sensor Benchmarks and Signal Quality

**Figure 6:**
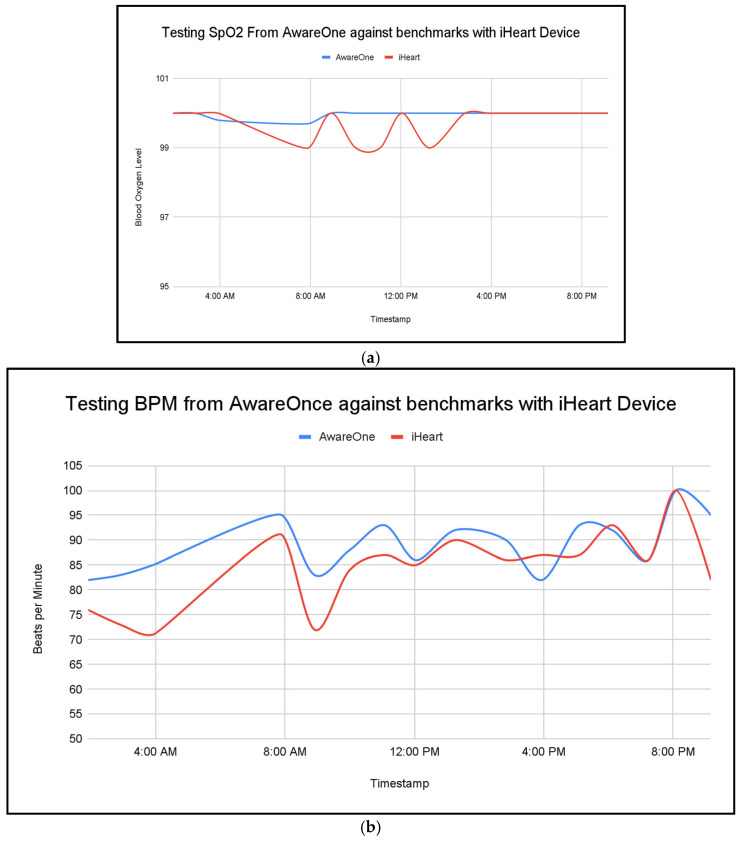
Comparison of SpO_2_ (**a**) and heart rate (**b**) readings between the proposed device and a commercial reference device over a one-day benchmark session, demonstrating mean absolute errors of 0.23 for SpO_2_ and 4.94 for BPM.

Against a commercial reference device, the proposed system achieved a mean absolute error of 0.23 for SpO_2_ and 4.94 for BPM in a one-day benchmark session, supporting basic physiological accuracy (Figure 6a,b).

**Figure 7:**
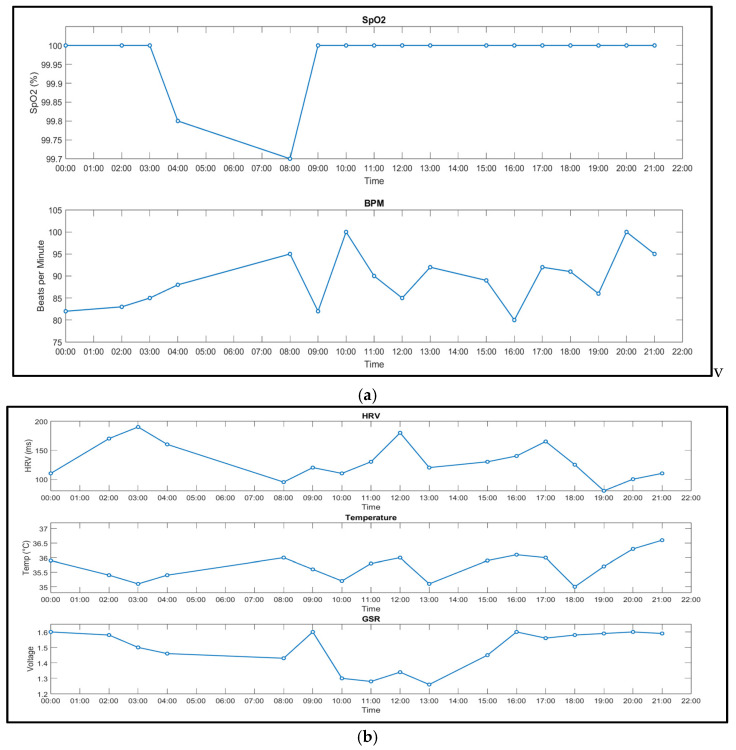
(**a**,**b**). Representative 24-h trace of the testing results. Patterns illustrate plausible autonomic variation and measurement stability. (**a**) Graph shows the SpO_2_ and beats per minute (BPM) values during rest and light activity. (**b**) Graph shows the heart rate variability (HRV), temperature, and galvanic skin response (GSR) values during rest and light activity.

The complete system was worn for a day, and the values of the biometrics were tracked. Time-aligned overlays show close tracking of heart rate and oxygen saturation under rest and during brief activity. The data represent segments that illustrate cohesive patterns and peaks during activity and rest throughout the day (Figure 7a,b).

### 3.2. Wearability and Data Yield

The proposed system and GSR device recorded a full day of physiological signals, demonstrating a stable and responsive performance. The SpO_2_ levels remained consistent throughout the day, reflecting stability in the readings aside from a minor dip in the early morning with a reading of 99.7%. Heart rate (BPM) has shown its ability to take a variety of readings, consistent with circadian arousal and physical activity. The system’s heart rate variability (HRV) exhibits some expected fluctuations, peaking at around 190 ms early in the morning and dipping below 100 ms in the evening, potentially correlating with increased stress or fatigue as the day progresses. The system’s skin temperature ranged between 34.88 °C and 36.6 °C, which is a healthy thermoregulation pattern, with a minor dip around 6 pm, then followed by a rise later in the evening. The GSR, which is measured via voltage changes, ranges from 1.2 V to 1.6 V, with the lower the voltage indicating higher arousal/stress and high voltage indicating relaxation. Comparing the HRV readings and GSR, it can be seen that there are some points throughout the day where the HRV and voltage are both low, which illustrates expected autonomic trends, for example, at 8 am and between 2:00 pm and 4:00 pm. Day-level coverage showed sustained collection. Figure 6 illustrates typical signal stability across HR, SpO_2_, temperature, and GSR during rest and light activity.

### 3.3. Fusion vs. Single-Sensor Performance

**Figure 8:**
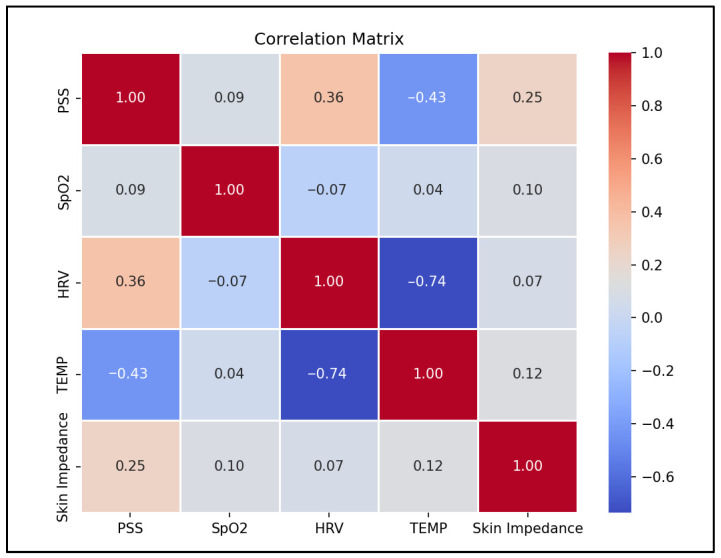
Correlation matrix showing the relationship between physiological features and simulated stress, demonstrating a strong negative correlation with temperature (−0.43) and a positive correlation with HRV (0.36).

**Figure 9:**
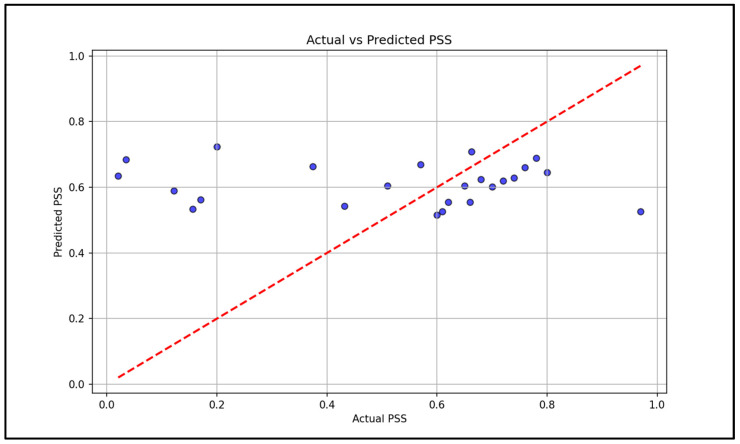
Scatter plot illustrating the performance of the support vector machine (SVM) model in predicting PSS scores, with a mean squared error (MSE) of 0.08, indicating a close alignment between actual and predicted values. The red dashed line represents perfect prediction, and the blue dots are the individual data points.

The system implements a machine learning algorithm to successfully identify stress by processing and analyzing the physiological data and fusing it with the active data to improve accuracy. In the current dataset, temperature showed the largest magnitude correlation with stress at −0.43, followed by heart rate variability (HRV) at 0.36, while SpO_2_ had a small correlation at 0.09 (Figure 8). An SVM model reached a mean squared error of 0.08 on stress prediction (Figure 9). This value represents the squared error between the predicted stress scores from the mid-level fusion model and the ground truth simulated labels. An error of 0.08 corresponds to a less than 10% deviation of the predicted scores from the normalized stress scale. These results indicate a satisfactory proof-of-concept evaluation. However, future work on a larger, real-world dataset is needed to confirm whether this performance is generalized.

## 4. Discussion

Stress triggers many physiological responses as it activates the body’s fight or flight response, and the non-invasive measurement of GSR, HRV, body temperature, and blood oxygen levels allows continuous monitoring that provides insights into the body’s response to stress. This work demonstrates the technical feasibility of integrating multi-sensor fusion with survey-based labels for stress monitoring. This approach joins standardized survey frameworks and passive wrist sensing to reduce gaps from either source alone. This fusion approach, particularly with the late-fusion survey data, is shown to improve the model’s accuracy and robustness. The fused model reached a mean squared error of 0.08, while single-day benchmarks showed consistency with the commercial reference device for SpO_2_ and BPM measurements. Temperature had the strongest association with simulated stress labels at −0.43, heart rate variability (HRV) showed a moderate association at 0.36, and SpO_2_ showed a small association at 0.09. These trends align with the expected physiology where the peripheral temperature often drops during acute stress and HRV changes with autonomic balance within the body [9,10,11,12].

Taken together, the signals indicate that a multi-model physiology provides complementary and strong information. While temperature and HRV contribute most to the prediction, GSR and SpO_2_ contribute more as an optical quality than as primary stress markers. The mid-level fusion approaches allow the encoders to learn appropriate scalings and nonlinearities of the features before concatenation. Late fusion with the self-reported questionnaires provides a simple advantage to incorporate user context when it is available, improving robustness as well when any single physiological feature/signal is compromised.

Within physiology, temperature-only and HRV models each may lose accuracy relative to a mid-level fusion and can underperform. This suggests that HRV and temperature data provide distinct and complementary data that the fused model can exploit. The correlation structure further supports this observation and will guide feature selection for future iterations.

Current and prior wrist systems typically rely only on HRV and activity to measure stress, limiting the robustness when motion artifacts occur or when a single marker is compromised or non-specific. The mid-level fusion on our device with the GSR, wrist temperature, SpO_2_, and HRV integrates complementary modalities, and the late fusion of the survey base labels provides context. Commercial wearables, such as the Apple Watch and Fitbit, provide HRV and its estimates but do not sense GSR, and they do not combine multi-model physiology with survey-based labels in a transparent fusion algorithm [4]. The wrist system model better aligns predictions with the user’s perception of stress. Current wrist devices’ late fusion depends on branching classifiers and Kalman filtering [4]. The wrist system uses its mid-level fusion followed by late fusion, allowing it to support more features and information before the final classification.

This work has several limitations. First, the evaluation used self-reported surveys and simulated labels, which limits generalizability. Second, the benchmark against commercial reference devices was conducted only over a single day, which does not capture the long-term drift or diverse conditions of sensor usability and wearability. Third, the correlation trends are exploratory and are/should not be interpreted as clinical findings. Future work will involve a larger-scale human subject validation under the appropriate ethical approval, including activity and posture normalization, and will evaluate personalization strategies.

## 5. Conclusions

The proposed system integrates wrist-based physiology with mid-level fusions of sensors and late fusion with the survey-based labels in a time-aligned workflow. Benchmarks showed close agreement with the device against a commercial reference device for SpO_2_ and BPM in a one-day session. In the representative tests, the fused model achieved an MSE of 0.08 and highlighted temperature and HRV as the most informative features. The results support the feasibility of daily stress monitoring on a wrist system.

## Data Availability

Data supporting the findings of this study are available from the corresponding author on reasonable request.
